# Effect of Acellular Amnion With Increased TGF-β and bFGF Levels on the Biological Behavior of Tenocytes

**DOI:** 10.3389/fbioe.2020.00446

**Published:** 2020-05-14

**Authors:** Rongli Sang, Yuanyuan Liu, Lingyu Kong, Ligang Qian, Chunjie Liu

**Affiliations:** ^1^Analytical and Testing Research Center, North China University of Science and Technology, Tangshan, China; ^2^Tangshan Vocational and Technical College, Tangshan, China; ^3^College of Integrated Chinese and Western Medicine, Hebei Medical University, Shijiazhuang, China; ^4^Department of Orthopedics, Affiliated Hospital of Hebei University of Engineering, Baoding, China; ^5^Department of Orthopedics, Tangshan Workers Hospital, Tangshan, China

**Keywords:** tendon adhesions, tenocytes, amnion, growth factor, collagen

## Abstract

The human amniotic membrane has been a subject for clinical and basic research for nearly 100 years, but weak rejection has been reported. The purpose of this research is to remove the cellular components of the amnion for eliminating its immune-inducing activity to the utmost extent. The amniotic membrane treated by acid removed the epithelial cell, fibroblast, and sponge layers and retained only the basal and dense layers. *In vitro*, biological effects of the new material on tenocytes were evaluated. The levels of transforming growth factor (TGF-β1), fibroblast growth factor (bFGF) proteins were measured. *In vivo*, the tendon injury model of chickens was constructed to observe effects on tendon adhesion and healing. The acellular amniotic membrane effectively removed the cell components of the amnion while retaining the fibrous reticular structure. Abundant collagen fibers enhanced the tensile strength of amnion, and a 3D porous structure provided enough 3D space structure for tenocyte growth. *In vitro*, acellular amnion resulted in the fast proliferation trend for tenocytes with relatively static properties by releasing TGF-β1 and bFGF. *In vivo*, the experiment revealed the mechanism of acellular amnion in promoting endogenous healing and barrier exogenous healing by evaluating tendon adhesion, biomechanical testing, and labeling fibroblasts/tendon cells and monocytes/macrophages with vimentin and CD68. The acellular amnion promotes endogenous healing and barrier exogenous healing by releasing the growth factors such as TGF-β1 and bFGF, thereby providing a new direction for the prevention and treatment of tendon adhesion.

## Introduction

Tendon injury caused by trauma is extremely common, and tendon adhesion to surrounding tissues is the most common complication after tendon repair. No effective solution is available for this complication to date (Jiang et al., 2014). More than 320,000 tendon injuries are caused by trauma and excessive exercise in the United States annually, and 30–40% of patients suffer from complications, such as limited joint movement. Adhesion after tendon repair has become an urgent clinical problem (Butler et al., 2008).

Tendon adhesion is closely related to tendon healing. Tenocytes on the surface and inside of the tendon are nourished by the surrounding synovial fluid and cytokines to stimulate the proliferation and secretion of collagen in repairing tendon. In the process of cell proliferation, tendon adheres to the surrounding tissues. Tenocytes are important activated cells during tendon healing and adhesion after tendon injury (Theiss et al., 2015). As the mediators of signal transduction between cells, growth factors can activate specific receptors on the cell surface, thereby leading to changes in gene transcription and cell behavior. Many growth factors are involved in regulating cellular response during tendon injury repair (Spanoudes et al., 2014; Tao et al., 2015). Growth factors, namely, transforming growth factor (TGF-β) and fibroblast growth factor (bFGF), promote tendon cell proliferation and stimulate collagen and fibronectin production (Qiu et al., 2014; Mehdizadeh et al., 2017). Therefore, mastering the molecular mechanism involved in tendon repair is necessary to improve tendon repair and prevent adhesion formation.

The human amniotic membrane has been a subject for clinical and basic research for nearly 100 years. As early as 1913, stem used an amniotic membrane for skin burn and ulcer wound transplantation (Amer et al., 2010; Hassan et al., 2017). However, the biological characteristics of the human amniotic membrane have obtained a completely new and in-depth understanding. Many growth factors in human amniotic membrane, such as TGF-β, bFGF, insulin-like growth factor-1 (IGF-1), vascular endothelial growth factor (VEGF), platelet-derived growth factor (PDGF), and tissue inhibitor of metalloproteinase, are available. These factors are important in regulating tendon injury repair and adhesion formation (Han et al., 1996; Kim et al., 2013; Vosdoganes et al., 2013; Fu et al., 2018). However, given that a fresh amniotic membrane is unsuitable for preservation and transportation, certain problems, such as immunogenicity, exist, thereby making it difficult to be widely used in clinical practice. Many scholars have applied fresh amniotic membranes to the clinical field, but weak rejection, such as local cyst formation after surgery, has been reported. Therefore, the removal of the cellular components of the amnion can eliminate its immune-inducing activity to the utmost extent (Lorenz et al., 2016).

After removal of the epithelial cell, the acellular amnion further reduces immunological rejection, making it a promising scaffold to support the attachment and proliferation of different cell types (Tang et al., 2018; Xue et al., 2018; Zhou et al., 2019). Minjuan et al. reported that the adipose derived mesenchymal stem cells seeded on the acellular amnion facilitated the healing of full-thickness skin wounds more effectively in nude mice (Minjuan et al., 2016). In a study by Chen et al. (2012) it was found that the acellular amnions were capable of providing a preferential environment for driving the osteogenic differentiation of humandental apical papilla cells. Acellular amnions have shown great potential for tissue regeneration. However, there have been no reports of tenocytes cultured on an acellular amnion. In this experiment, the amniotic membrane treated by acid removed the epithelial cell, fibroblast, and sponge layers and retained only the basal and dense layers. Acellular amniotic membrane is soft, biocompatible, and has no evident immune induction activity. Acellular amniotic membrane also retains collagen scaffolds and growth factors, such as TGF-β and bFGF, which promote cell proliferation. This membrane is a natural medium for cell culture. The effects of acellular amniotic membrane on the biological behavior of tenocytes *in vivo* and *in vitro* were observed, and the molecular mechanism leading to tendon adhesion was explored.

## Materials and Methods

### Materials

#### Isolation and Culture of Tenocytes

All protocols of the animals were approved by the Ethics Board of the Third Hospital of Hebei Medical University, and the subject was conducted in accordance with the institutional guidelines for the care and treatment of animals.

Leghorn chickens aged 6 months and weighing 1.0–1.5 kg were provided by the Laboratory Animal Center of Hebei Medical University. Tendon tissues were harvested under sterile conditions from the flexor digitorum profundus tendon (FDP) of adult Leghorn chickens. The harvested tendons were immediately placed in a 50 ml tube containing 20 ml of PBS along with 100 U/ml of penicillin and 100 U/ml of streptomycin. The peritendinous tissue was dissected under a microscope. Then, the tendons were chopped into small pieces with the dimension of approximately 1 mm × 1 mm × 1 mm and washed three times with PBS. The chopped tendons were digested for 1 h with 0.25% trypsinase and 0.1% collagenase at 37°C and vibrated every 10 min during digestion. The mixture was filtered to remove tissue residues through a sterile nylon mesh, and the filtrate was centrifuged at 800 r/min for 10 min. The supernatant was removed, and the cell was resuspended in DMEM containing 10% fetal bovine serum (FBS), 100 U/ml penicillin, 292 mg/ml L-glutamine, 100 U/ml streptomycin, and 50 mg/ml ascorbic acid. Cells were seeded onto 10 cm culture dishes and then placed in an incubator. The petri dishes were cultured at 37°C in 5% CO_2_ and 95% air incubators. Then, the liquid was changed 2–3 times a week, but only half of the liquid was changed each time. When tendon cells proliferated and fused to 80%, they were passaged. During subculturing, the original culture medium was discarded, the necrotic cells and the residual culture medium were washed out with PBS solution, 1 ml of 0.25% trypsin was added, and the culture dish was placed in the incubator for 3 min. Under an inverted microscope, adherent cells were flaked off from the petri dish and retracted into a round shape. A fresh culture medium was added to blow into a uniform cell suspension. Then, they were passed on to a culture flask at the ratio of 1:2 and placed in a culture box. Three to five generations were used in the experimental study. Then, tenocytes were collected by 0.25% trypsin digestion to be used in the following procedures.

#### Acellular Amniotic Membranes

A fresh amniotic membrane was provided by the Department of Obstetrics and Gynecology of the Third Hospital of Hebei Medical University. With the consent of the pregnant women, serological tests were performed on the pregnant women, and the results showed that they were negative for HBV, HCV, HIV, syphilis, and gonorrhea. After the blunt separation between amniotic membrane and chorion, a smooth, translucent amniotic membrane was obtained and then soaked in a balanced salt solution containing 50 μg/ml of penicillin and 50 μg/ml of streptomycin for 20 min, placed in DMEM medium, stored in a 4°C refrigerator, and used within 24 h.

The fresh amniotic membrane was washed three times with PBS containing 50 μg/ml of penicillin and 50 μg/ml of streptomycin, removed the spongy layer, and cut into 1.0 cm × 0.5 cm pieces. The epithelial cells were dislodged by the culture in 0.05% ethylenediaminetetraacetic acid at 37°C for 2 h and wiped off under a microscope with a cell scraper. The acellular amniotic membrane was preserved in an aluminum foil film bag that was vacuum-tightened and sterilized with ethylene oxide for 6 h.

#### Histological Analysis and DNA Quantification

he native amnion and acellular amnion were fixed in 10% formaldehyde, dehydrated in graded ethanol and xylene, and subsequently embedded in paraffin. Sections of 5 mm were cut and were stained with hematoxylin and eosin (H&E). The specimens were fixed in 2.5% glutaraldehyde and then dehydrated with a graded ethanol series. After gradient dehydration, the dried specimens were sputter-coated with gold before being examined under scanning electron microscopy (SEM). The remaining cellular DNA in the acellular amnion was measured using Quant-iT PicoGreen dsDNA Assay Kit (Invitrogen Inc., United States). Fluorescence value was detected by multi-functional microplate reader (Tecan Infinite F200 Pro, Switzerland). The standard curve was drawn by testing the DNA content of HS reagent in Quant-iTTM.

### *In vitro* Cell Experiment

#### Nuclear Fluorescence Staining

The acellular amniotic membrane was placed in a 24-well plate, and the blank control group was created with the same treatment except for the acellular amnion. Tenocytes were inoculated on the material at a density of 2 × 10^4^ cells/cm^2^ and cultured in the DMEM medium containing 10% FBS and 1% biantibody for 2 and 5 days before fluorescence staining. The acellular amniotic membrane was rinsed three times with PBS, fixed with 4% polyformaldehyde solution for 10 min, permeated with 0.1% Triton X-100 solution 200 μl for another 10 min, and sealed with 5% goat serum for 45 min. Chroest (Shenzhen Runtai Technology Co., Ltd., China) was added to stain the cell nuclei. The nuclei were placed under a fluorescence microscope, and the pictures were taken.

#### Collagen I and Fibronectin Fluorescence Staining

Collagen I antibody and fibronectin antibody (Wuhan Boster Biological Technology Co., Ltd., China) were diluted at a ratio of 100:1 with the addition of PBS solution, while the control group was added with PBS solution and then placed in a refrigerator at 4°C overnight. After rewarming for 30 min at room temperature, the primary antibody was aspirated, and fluorescent FITC-labeled collagen I and fibronectin secondary antibody (Wuhan Boster Biological Technology Co., Ltd., China) were diluted with the addition of PBS at the ratio of 40:1 and evaluated under a fluorescence microscope. Microscopy images were acquired using an Olympus IX73P2F with the same light exposure times. Images were analyzed using Image Pro Plus software 6.0 (Media Cybernetics, United States). Five images per treatment were captured: one sampling image was taken from each of the four quadrants and one in the center of the well. Adjustments of brightness and contrast were applied equally across each image.

#### Cell Proliferation Assessment

The acellular amniotic membrane was placed in a 24-well plate, and the blank control group was created. Tenocytes were inoculated at the density of 5 × 10^3^ cells/cm^2^ and cultured in the DMEM medium containing 10% FBS and 1% biantibody. Cell viability was detected by CCK-8 method after the digestion of cells from different materials with 0.25% trypsin on the 1st, 3rd, 5th, 7th, and 10th day, respectively. The absorbance of each group was determined by an enzyme-labeling instrument (measuring wavelength of 450 nm). The final absorbance value was obtained by subtracting the absorbance of the blank group. The growth curve was plotted with the number of culture days on the horizontal axis and the absorbance value on the vertical axis.

#### Western Blot Analysis

Cells were cultured and grouped in the same way. Cells were collected on the 7th day after culture. Proteins were extracted with cell lysate (20 mmol/l of HEPES, pH of 7.2, 1% Triton X-100, 10% triacylglycerol, 150 mmol/l of NaCl, 1 mmol/l of Na_3_OV_4_, 10 mg/l of leucostatin, and 1 mmol/l of PMSF). The protein concentration of the cell was measured using the BCA protein assay kit (Beijing Solarbio Science and Technology Co., Ltd., China). The samples were electrophoresed through a 8% SDS-PAGE gel and then transferred onto a PVDF membrane. After being blocked with 5% non-fat milk, the membranes were incubated with antibodies against collagen I, fibronectin, TGF-β1, bFGF (Abcam, United States), and β-actin (Beijing Bioss Biotechnology Co., Ltd., China) overnight at 4°C. After being washed, the membranes were incubated with the secondary antibodies (Abcam, United States) for 1 h. The membrane was washed three times with TBST buffer (50 mm Tris–HCl, 100 mm NaCl, and 0.1% Tween 20, pH of 7.4) and scanned with the Odyssey Fc System (LICOR, United States).

### Preliminary Animal Study

Tendon injury models were established in 30 chickens on the ipsilateral third toe. The experimental animals were anesthetized with ketamine at a dose of 25 mg/kg via intramuscular injection. The flexor tendon was exposed through a longitudinal incision of approximately 1.5 cm at the proximal interphalangeal joint of the third toe. A longitudinal incision of approximately 1.5 cm was created at the proximal interphalangeal joint of the third toe, and the flexor digitorum tendon was exposed. Partial flexor digitorum superficialis tendon was excised, and FDP was free and cut off. Then, the severed tendon was sutured with 5–0 non-invasive “8” suture. In the amniotic membrane group, an appropriate size of acellular amniotic membrane was wrapped around the place where the tendon was stitched, and the edge was sutured to fix. The control group did not undergo any other treatment. After the operation, the experimental animals were reared in cages, and their basic conditions of the were observed regularly. Sterile dressing was applied to the incision, and the animals were supplied with feed containing oxytetracycline for 3 days after operation. The suture was removed 2 weeks after the surgery, and the cast was fixed for 4 weeks.

#### Macroscopic and Microscopic Evaluation

At the 2nd, 4th, and 6th week after the operation, 5 chickens in each group were killed via excessive ketamine injection. The third toe was extracted to observe the adhesion between the tendon and the surrounding tissues. Tendon adhesion (macroscopic and microscopic) was evaluated according to the criteria defined by Tang et al., 2018 (Supplementary Tables S1A,B).

#### Immunohistochemical Evaluation

Tendons were harvested at each time point, fixed with 4% polyformaldehyde, embedded in paraffin, and sectioned continuously (4 μm). Vimentin antibody (Abcam, United States), CD68 antibody (ARP, United States). The known positive piece was used as the positive control, and PBS was used as the negative control instead of the primary antibody. The field of vision was randomly selected for each slice under a 400-fold microscope. Vimentin immunostaining positive substance was brown, which was mainly expressed in the cytoplasm and nucleus, and CD68 was located in the cell membrane and cytoplasm.

Slides were evaluated and scored by two experienced pathologists (K.L.Y and R.L.S). The immunohistochemical score (IHS) is based on the German Immuno Reactive score. The IHS is calculated by combining an estimate of the percentage of immunoreactive cells (quantity score) with an estimate of the staining intensity (staining intensity score). as follows: no staining is scored as 0, 1–10% of cells stained scored as 1, 11–50% as 2, 51–80% as 3, and 81–100% as 4. Staining intensity is rated on a scale of 0 to 3, with 0 = negative; 1 = weak; 2 = moderate, and 3 = strong. The raw data was converted to the IHS by multiplying the quantity and staining intensity scores. An IHS score of 8–12 was considered high expression, 0–7 was considered low expression (Soslow et al., 2000).

#### Biomechanical Evaluation

At the 2nd, 4th, and 6th week after the operation, the metacarpophalangeal joints were severed, and the sliding distance, total flexion angle, and maximal tensile strength were measured using a biomechanical experimental machine. The proximal phalanges of toes were fixed on the experimental machine, and the retained FDP was pulled by 1 N force, thereby gradually increasing the traction force to 10 N. The length of the tendon that was pulled out from the tendon sheath was marked and measured. The total flexion angles (the sum angles of PIP and DIP) were measured and calculated with a goniometer. The two ends of FDP were fixed on the biomechanical testing machine, and the tension adjusted. Then, the two ends moved at the speed of 20 mm/N until the tendon ruptured, and the maximum tensile rupture strength of the tendon was recorded synchronously.

### Statistical Analysis

SPSS 24.0 and GraphPad Prism7 statistical software were used for statistical analysis. The results were tested for variance normality and homogeneity. If the variance was homogeneous, then *t*-test and non-parametric test were used. Mann–Whitney test was used to evaluate the tendon adhesion. The immunohistochemical values were calculated using the Fisher exact method. *P* < 0.05 was considered statistically significant.

## Results

### Histological Analysis and DNA Quantification

**FIGURE 1:**
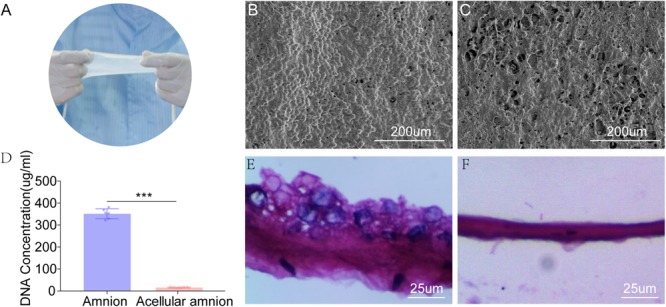
The appearance of fresh amniotic membranes **(A)**. The epithelial cell layer of fresh amniotic membranes **(B)** and the histoarchitecture of the acellular amnion **(C)** observed by SEM. The cell structure was not detected and the intimal surface was rougher than that of the native amnion. **(D)** The remaining cellular DNA in the amnion and acellular amnion was measured using Quant-iT PicoGreen dsDNA Assay Kit. The total DNA content (351.5 ± 9.23 ug/mL) decreased significantly after the decellularization treatment when compared to the acellular amnion (15.66 ± 0.82 ug/mL). The epithelial cell layer of native amniotic membranes **(E)**. Hematoxylin-eosin (H&E) staining confirmed complete removal of epithelial cells **(F)**. ***p < 0.001.

The SEM images revealed that the histoarchitecture of the acellular amnion was maintained the same as the native amnion, the cell structure was not detected and the intimal surface was rougher than that of the native amnion. The total DNA content (351.5 ± 9.23 ug/ml) decreased significantly after the decellularization treatment when compared to the acellular amnion (15.66 ± 0.82 ug/ml). Compared with the native amnion, the cellular components were not detected by (H&E) staining in the acellular amnion (Figure 1).

### Tenocyte Growth and Proliferation

**FIGURE 2:**
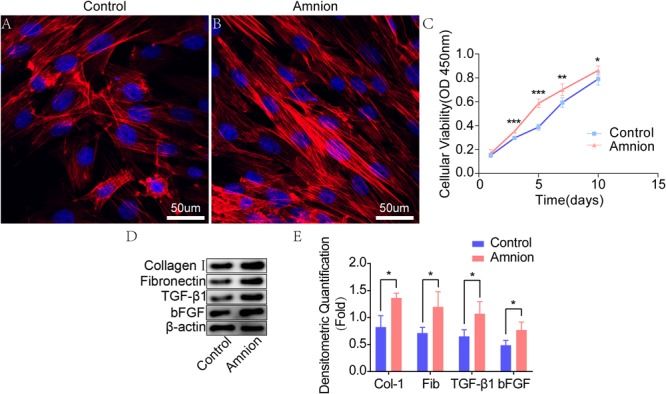
Fluorescence images of tenocytes after 5 days of culture on the surface of a culture plate (control group) and acellular amniotic membrane (amnion group). Tenocytes presented a clear cytoskeleton, good biocompatibility with acellular amniotic membrane, and even distribution on the surface of the materials, showing better growth activity than the control group **(A,B)**. Cell viability was measured by CCK-8, and the proliferation curve of tenocytes in the control and amniotic membrane groups was drawn **(C)**. Western blot assay for collagen I, fibronectin, TGF-β1, and bFGF expression in the tenocytes of the control and amnion groups for 1 week **(D,E)**. *p < 0.05; **p < 0.01; and ***p < 0.001.

The tenocytes in the control and amniotic membrane groups had clear outline, strong stereoscopic sense, long spindle shape, decreased cytoplasm, round or oval nucleus, and inevident nucleolus. The growth of tendon cells in the control group was slower than that of tendon cells in the amniotic membrane group. Growth and proliferation curve: After being cultured for 1, 3, 5, 7, and 10 days, tenocytes were counted using the CCK-8 method and gradually increased with time. The difference in the activity of tenocytes between the two groups after being cultured for 1 day was insignificant. After 3, 5, and 7 days of culture, the growth of acellular amniotic membrane cells was faster, and the activity was higher than those of the control group (*P* < 0.05). The cell growth curve showed that the growth trend of the amniotic membrane slowed down after 5 days, while that of the control accelerated after 5 days (Figures 2A–C).

### Western Blot

Western blot results showed that the collagen I, fibronectin, TGF-β1, and bFGF expression levels in the amniotic membrane group were significantly higher than those in the control group (Figures 2D,E).

### Fluorescence Staining of Tenocytes

**FIGURE 3:**
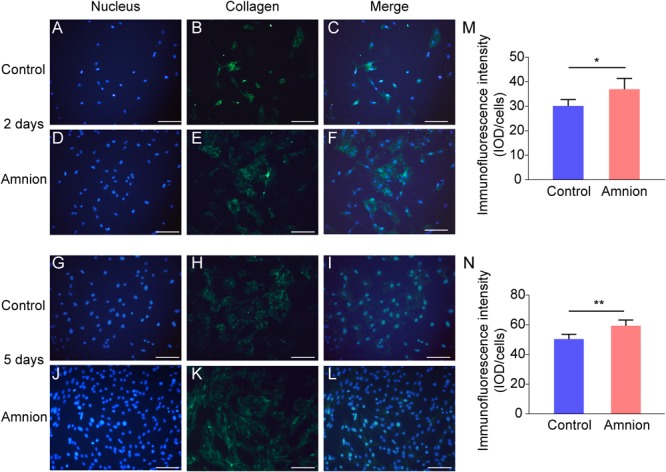
Representative immunofluorescence images of collagen I. Fluorescent micrographs of tenocytes after 2 and 5 days of culture on the surface of a culture plate and acellular amniotic membrane. Tenocyte nucleus shape observed under a fluorescence microscope **(A,D,G,J)**. Collagen I presented positive after the fluorescent FITC mark was observed under a fluorescence microscope **(B,E,H,K)**. Tenocyte nucleus and collagen I merging **(C,F,I,L)**. The corresponding semi quantitative analysis of collagen fluorescence intensity in panels **(M,N)** (scale bar = 50 um, *n* = 5, *P < 0.05 and **P < 0.01).

**FIGURE 4:**
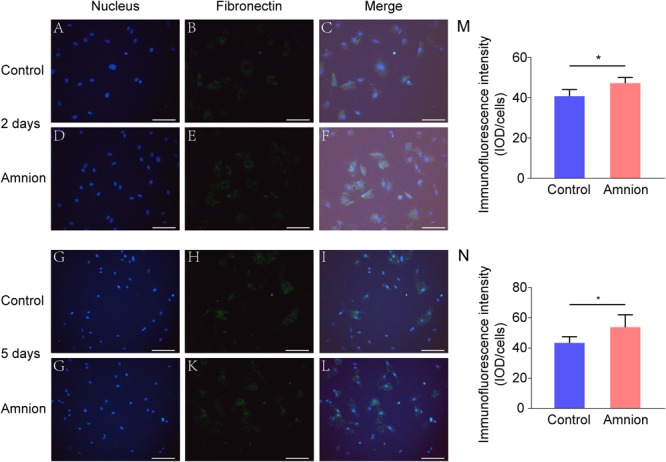
Representative immunofluorescence images of fibronectin. Fluorescent micrographs of tenocytes after 2 and 5 days of culture on the surface of a culture plate and acellular amniotic membrane. Tenocyte nucleus shape observed under a fluorescence microscope **(A,D,G,J)**. Fibronectin presented positive after the fluorescent FITC mark was observed under a fluorescence microscope **(B,E,H,K)**. Tenocyte nucleus and fibronectin merging **(C,F,I,L)**. The corresponding semi quantitative analysis of fibronectin fluorescence intensity in panels **(M,N)** (scale bar = 50 um, *n* = 5, *P < 0.05).

At the same time, the number of nuclei was higher, and the area of collagen I and fibronectin was larger in the amniotic membrane group than in the control group (Figures 3, 4).

### Tendon Adhesion Evaluation

**FIGURE 5:**
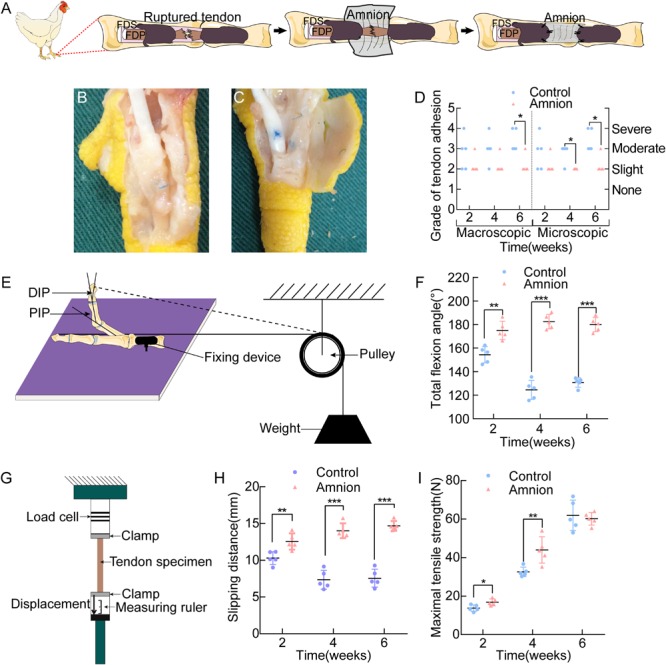
**(A)** Operative technique for the application of the acellular amnion allograft. Establishment of flexor digitorum profundus tendon model in chickens. The tendon has been sutured, and the membrane is placed between the flexor tendons. The membrane is wrapped around the FDP tendon and fixed to the remaining tendon sheath. General observation of the control group **(B)** and amnion group **(C)** on the 4th week after the operation. **(D)** The macroscopic and microscopic evaluation of adhesion on the 2nd, 4th, and 6th week after the operation. Scheme of the total flexion angle measurement **(E)** and the results of the total flexion angle **(F)**. Scheme of the sliding distance, the maximal tensile strength **(G)** and the results of the sliding distance **(H)**, the maximal tensile strength **(I)** between the two groups on the 2nd, 4th, and 6th week after the operation. *p < 0.05; **p < 0.01; and ***p < 0.001.

At 2 weeks after the operation, the tendon anastomosis was enlarged, and the acellular amniotic membrane was blurred, recognizable, and non-adherent to the surrounding tissues. In the control group, additional fibrous tissue grew into the tendon anastomosis area. At 4 weeks after the operation, the amniotic membrane in the amniotic membrane group was difficult to identify, and the tendon anastomosis healed well. In the control group, the tendons and peritendinous tissues were connected with many fibrous tissues, thereby increasing the difficulty of dissection. At 6 weeks after the operation, the tendons in the amniotic membrane group healed well without adhesion, which was similar to the normal structure. In the control group, the peritendinous tissue adhered considerably to the tendon anastomosis, and the tendons showed difficultly in forming an effective sliding (Figures 5A–C).

Adhesion was more or less present in all groups. The differences between the acellular amniotic membrane and control groups in the macroscopic evaluation of adhesion at the 6th week after operation and in the microscopic evaluation of adhesion at the 4th and 6th week after the operation were statistically insignificant (*P* < 0.025, Figure 5D).

### Biomechanical Evaluation

The differences in the total flexion angle of the toes (Figures 5E,F) and sliding distance (Figures 5G,H) between the two groups at each time point after the operation were significant (*P* < 0.05).

Maximum tensile breaking strength of the tendon: At the 2nd and 4th week after the operation, the maximal tensile strength of the acellular amniotic membrane group was higher than that of the control group (*P* < 0.05). At the 6th week after the surgery, the difference in the maximum tensile rupture strength of the tendon was insignificant (*P* > 0.05, Figure 5I).

### Immunohistochemical Evaluation

**FIGURE 6:**
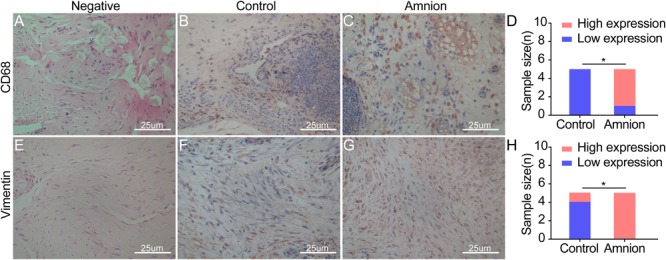
Exemplary images of inflammatory cell and fibroblast infiltration visualized by immunohistological CD68 (monocytes/macrophages) **(A–C)** and vimentin (fibroblasts/tenocytes) **(E–G)** staining in ruptured tendons between the control group and amnion group at the 2nd week after operation. The CD68 and vimentin of the acellular amniotic membrane group were higher than that of the control group **(D,H)**. High expression of CD68 and vimentin in the amnion group meaned that a large number of monocytes/macrophages infiltrated, neovascularization increased, and fibroblasts/tenocytes proliferated rapidly. *p < 0.05.

Microscopic observation showed that CD68 expression in the amniotic membrane group was significantly increased and more concentrated than that in the control group 2 weeks after the operation, but it gradually decreased 4 weeks after operation. The difference between the amniotic membrane and control groups at 6 weeks was insignificant (Figures 6A–D). At each time point, vimentin protein expression in the amniotic membrane group was higher than that in the control group. Vimentin protein expression in the amniotic membrane group was stratified and compact, while the vimentin protein distribution in the control group was disordered (Figures 6E–H).

## Discussion

**FIGURE 7:**
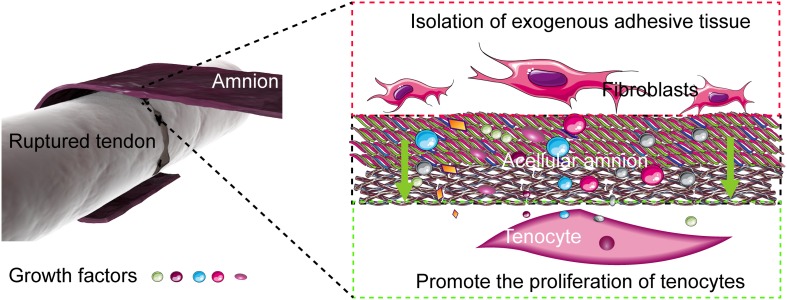
Mechanism of the acellular amniotic membrane that promotes endogenous healing of tendon and prevents exogenous adhesion. The acellular amnion contains several active factors, such as TGF-β1, bFGF. These factors are released to the tendon repair area, which promotes the proliferation of tenocytes and the synthesis of collagen. The acellular amnion also functions as a barrier to fibroblasts and other exogenous tissue invasion.

Restrictive adhesion, which seriously affects the recovery of the hand function, often occurs after tendon repair. Restrictive adhesion has been a major problem in hand surgery. At present, two mechanisms of tendon healing after tendon injury, namely, exogenous and endogenous healing, exist. Exogenous healing is the invasion of fibroblasts and inflammatory cells from tendon sheath and peritendinous synovium into the healing site, which eventually leads to scar formation and tendon adhesion to the surrounding tissues. In exogenous healing, the migration of fibroblasts, the deposition of collagen matrix, and the contraction of fibrous tissue are the main mechanisms of adhesion formation. Endogenous healing is mediated by tenocytes within the tendon, which mainly promotes the healing of broken tendon ends. Therefore, knowing how to control exogenous healing and promote endogenous healing is important to reduce tendon adhesion and the direction of most scholars in solving the problem of tendon adhesion and improve the quality of tendon healing (Zhang et al., 2014; Figure 7).

Familiarizing with the reaction process after tendon injury has an important guiding significance for the prevention and treatment of tendon adhesion. The repair process after tendon injury mainly includes acute inflammation, proliferative, and remodeling phases. During the inflammation stage, cell membrane destruction and cell matrix release are mainly mediated by cytokines, such as bFGF, TGF-β1, IGF-1, PDGF, and VEGF, and inflammatory factors, such as IL-1, IL-6, and TNF-α. The main clinical manifestations are tissue edema, fibrin exudation, and inflammatory cell production (Backman et al., 2014; Snedeker and Foolen, 2017; Sugg et al., 2018). Subsequently, tenocytes and fibroblasts proliferate rapidly under the drive of growth factors, such as TGF-β1, bFGF, IGF-1, PDGF, and VEGF. Inflammatory cells remove damaged tissues and gradually form fibronectin, collagen, and other extracellular matrix components (proteoglycan and extracellular matrix glycoprotein) in a disordered manner, thereby eventually forming a granulated tissue. At the remodeling stage, collagen I begins to remodel the structure and form orderly fibers, thereby inducing the repaired tendons to have a certain mechanical load capacity (Paredes and Andarawis-Puri, 2016).

The main approach to reduce the exogenous healing of tendons is to use a mechanical barrier of biological or non-biological materials. Many specific materials are available for research. Considering the different physical and chemical properties of materials, their mechanisms and clinical effects are also different. Early non-biological materials, such as silica gel, gold foil, and autologous fascia, have many limitations and have been gradually eliminated. Scholars have turned to absorbable macromolecule compounds, such as hyaluronic acid (HA), polylactic acid (PLA), polyhexanoic acid (PGA), and polydextral lactic acid. Moro-oka injects HA into the intrathecal and tendon anastomosis sites to promote tendon healing by alleviating inflammation and promote the links between epidural and endotendinous cells (Moro-oka et al., 2000). However, considering the possible adverse effects, such as local pain and increased risk of peripheral inflammation, Intergel^®^ was withdrawn for further investigation in the United States market in 2003 (Metwally et al., 2008).

Polyhexanoic acid and PLA materials are widely used in clinical setting because of their good biocompatibility. However, PLA degrades *in vivo* for >3 months, while PGA degrades into lactic acid *in vivo* after 10 months to 4 years. The degradation of these macromolecule compounds *in vivo* would lead to matrix degradation and cell apoptosis. With the development of tendon repair research, although this non-biological material membrane can isolate tissues and prevent adhesion, it increases the possibility of tendon necrosis and permanent foreign body residue due to lack of permeability, thereby hindering nutrient penetration (Li et al., 2017).

Amniotic membrane is a kind of natural macromolecule biomaterial with the thickness of approximately 0.02–0.5 mm. Amniotic membrane does not contain blood vessels, nerves, and lymph. Under the microscope, the amniotic membrane is divided into five layers, namely, epithelial layer, basement membrane, dense layer, fibroblast layer, and spongy layer. Amniotic membrane contains collagen, proteoglycan, and glycoprotein. Amniotic membrane also expresses many growth factors, such as bFGF, TGF-β1, IGF-1, PDGF, and VEGF, which can provide sufficient nutrients for the proliferation of tenocytes and exert the function of immune regulation, thereby promoting tendon repair (Hortensius et al., 2018).

Growth factors are natural proteins that exist in organisms and participate in biological processes, such as cell growth, development, and repair. Similarly, many cytokines are involved in tendon development, maturation, and repair. For example, bFGF can promote the proliferation and differentiation of mesoderm and neuroectoderm cells. Hence, bFGF is considered as a broad-spectrum mitogen. The ability of bFGF to promote the proliferation of tenocytes and the healing of tendon has been confirmed by many studies. Hankemeier et al. (2005) found that exogenous low-dose bFGF can promote the proliferation of bone marrow mesenchymal stem cells and significantly increase the expression of ligament-specific extracellular matrices, such as collagen I and fibronectin mRNA. Another important biological function of bFGF is to promote angiogenesis, which degrades the extracellular matrix in the wound area and extends the capillaries to the wound area (Ye et al., 2018). Revascularization is a key step in tendon repair to ensure adequate nutritional supply and eliminate metabolites, thereby maintaining the vitality of tendon cells and promoting tissue remodeling.

Transforming growth factor-β1 can regulate tenocyte proliferation and differentiation and promote collagen I production. Mechanical stimulation maintains the *Sex* gene expression through the signal pathway mediated by TGF-β1/Smad2/3 (Vermeulen et al., 2019). Subramanian et al. reported that TGF-β1 transfection *in vivo* after tendon injury can remarkably improve the quality of the repaired tendon through histological observation (Subramanian et al., 2018). The classical view is that bFGF stimulates the proliferation of tenocytes and fibroblasts through the RAS–MEK–ERK pathway (Katz et al., 2007), while TGF-β1 stimulates the secretion of collagen and other products through the SMAD pathway (Heldin et al., 1997; Moustakas et al., 2001).

In the present experiment, the acellular amniotic membrane treated by acid was a semipermeable membrane that effectively removed the cell components of the amnion while retaining the fibrous reticular structure of the basement membrane and the dense layer. Abundant collagen fibers enhanced the tensile strength of amnion, and a 3D porous structure provided enough 3D space structure for tenocyte growth.

The methods of tenocyte culture *in vitro* are becoming increasingly mature, which further promotes the in-depth study of preventing tendon adhesion. Tenocytes are a special type of fibroblast belonging to terminal differentiation cells. The tenocytes in a tendon tissue are few, and they are arranged vertically in located between collagen fibers. Generally, tenocytes stop growing *in vivo*, and the isolation and culture *in vitro* are relatively difficult. In the present experiment, the tenocyte growth rate was relatively slow, and growth curve was smooth, thereby showing relatively static characteristics. After acellular treatment, the amnion removed the immunogenicity of biomaterials while retaining the original growth factors, such as TGF-β1 and bFGF, and the 3D space structure necessary for cell growth. It is a natural extracellular matrix that is close to the composition of the connective tissue. Thus, it has a strong affinity for tendon cells.

With the continuous improvement of cell and molecular biology research, the changes in the reactivity of fibronectin during tendon injury and repair have attracted considerable interest. Welch believed that during tendon repair, tendon cells and fibroblasts synthesize a large amount of fibronectin, which is secreted into the cell surface and then extended between cells, thereby forming a connection with fibronectin receptors on the surface of adjacent cells and fibrous components in the interstitium to reinforce the firmness of the connective tissue horizontally (Welch et al., 1990). Meanwhile, Jozsa et al. (1989) observed the distribution of fibronectin in a tendon tissue and found that fibronectin is negative in a normal tendon tissue but positive in a ruptured tendon tissue. Similar phenomena were observed in cell proliferation experiments *in vitro*. Thus, fibronectin is detected in and around newly formed and proliferated tendon cells (Jozsa et al., 1989; Branford et al., 2012).

Vimentin is a marker of interstitial cells and an important component of the cytoskeleton. Vimentin helps cells resist various external pressures, including mechanical pressures, viruses, and other adverse factors for cell survival. Vimentin also plays an important role in cell adhesion, migration, and signal transduction (Coulombe and Wong, 2004; Toivola et al., 2005) and is often used as a developmental indicator of cells and tissues (Ivaska et al., 2007). CD68 is a cytoplasmic glycoprotein that is related to lysosome granules and a specific marker of monocytes. Monocytes often exist in blood vessels (Szabo and Younger, 1990; Dakin et al., 2018). During tendon repair, fibroblasts/tendon cells and monocytes/macrophages were labeled with vimentin and CD68 by immunohistochemical method (Klatte-Schulz et al., 2018; Pellegrini et al., 2020). At an early stage of tendon injury, a large number of monocytes/macrophages infiltrated, neovascularization increased, and fibroblasts and tendon cells proliferated rapidly. The vimentin protein and CD68 expression levels in the acellular amniotic membrane group was higher than that in the control group. The distribution of vimentin protein was also layered and compact, while that of vimentin protein in the control group was disorderly. The defects or disordered arrangement of vimentin can lead to the decline of tenocyte and fibroblast migration ability (Eckes et al., 2000), poor tendon healing, abnormal mechanical properties (Henrion et al., 1997), and the decrease in vascular endothelial integrity (Nieminen et al., 2006).

Although the mechanism of tendon repair is well understood, the repair process after tendon injury is a multi-cell, multi-factor-involved dynamic physiological process, and it is difficult to produce a significant effect by supplementing a single growth factor. Moreover, the application of separate growth factors also has shortcomings such as short half-life, single effect, and high price. Researchers selectively activate PRP to release different growth factors or add different exogenous growth factors, which can produce different tendon repair effects (Rajpar and Barrett, 2019; Zhang et al., 2019). Due to its own source, the amniotic membrane contains the promoting factors and inhibitory factors which are internal proportions of the body, rather than random mixing. However, the optimal ratio for promoting tendon healing remains to be confirmed.

## Conclusion

In this experiment, the acellular amnion treated by acid effectively removed the cellular components of amnion while retaining the fibrous reticular structure of basement membrane and dense layer and retaining the growth factors, such as TGF-β1 and bFGF, and the 3D space structure necessary for cell growth, which is a natural extracellular matrix. *In vitro*, acellular amnion resulted in the fast proliferation trend for tenocytes with relatively static properties by releasing TGF-β1 and bFGF. *In vivo*, the experiment revealed the mechanism of acellular amnion in promoting endogenous healing and barrier exogenous healing by evaluating tendon adhesion, biomechanical testing, and labeling fibroblasts/tendon cells and monocytes/macrophages with vimentin and CD68. Hence, the acellular amnion promotes endogenous healing and barrier exogenous healing by releasing the growth factors such as TGF-β1 and bFGF, thereby providing a new direction for the prevention and treatment of tendon adhesion.

## Data Availability Statement

The datasets analyzed in this article are not publicly available. Requests to access the datasets should be directed to CL.

## Ethics Statement

The studies involving human participants were reviewed and approved by the Ethics Board of the Third Hospital of Hebei Medical University. The patients/participants provided their written informed consent to participate in this study. The animal study was reviewed and approved by the Ethics Board of the Third Hospital of Hebei Medical University.

## Author Contributions

CL proposed the experiment concept and designed the study. RS collected the data. LQ, YL, and LK analyzed the data and prepared the figure. RS wrote the main manuscript text. All authors reviewed the manuscript and provided critical revision.

## Conflict of Interest

The authors declare that the research was conducted in the absence of any commercial or financial relationships that could be construed as a potential conflict of interest.
