# Supplementary material for: Effect of Acellular Amnion With Increased TGF-β and bFGF Levels on the Biological Behavior of Tenocytes
Source: Front Bioeng Biotechnol. 2020 May 14;8:446. doi: 10.3389/fbioe.2020.00446 (PMC7240037; doi:10.3389/fbioe.2020.00446)
Supplement: Supplementary file 1 [file Table_1.DOCX]

Supplementary Material

1. Tendon adhesion (macroscopic and microscopic) was evaluated due to the criteria defined by Tang et al. (Tables 1A and 1B). Tang JB, Shi D, Zhang QG.Biomechanical and histo logic evaluation of tendon sheath management, Journal of Hand Surgery, 1996, 21(5):900–908.doi: 10.1016/S0363-5023(96)80212-7.

Table 1: Evaluation of adhesions. A. Criteria described by Tang et al. for macroscopic evaluation of adhesions.

| **A** | Points | Adhesion appearance |
| --- | --- | --- |
| Length | 0 | No adhesion |
|  | 1 | Localized, <10mm longitudinal |
|  | 2 | 10–15mm |
|  | 3 | Intense, >15mm |
| Characteristics | 0 | No adhesion |
|  | 1 | Loose, elastic, and mobile |
|  | 2 | Of average thickness and mobile |
|  | 3 | Thick, hard, and immobile |
| Grading | 0 | No adhesion |
|  | 1 | Mild adhesion |
|  | 2 | Moderate adhesion |
|  | 3 | Advanced stage adhesion |

B. Criteria described by Tang et al. for microscopic evaluation of adhesions.

| **B** Points | Features of adhesion |
| --- | --- |
|  | Quantity |
| 0 | No apparent adhesions |
| 1 | A number of scattered filaments |
| 2 | A large number of filaments |
| 3 | Countless filaments |
|  | Quality |
| 0 | No apparent adhesions |
| 1 | Regular, elongated, fine, and filamentous |
| 2 | Irregular, mixed, shortened, and filamentous |
| 3 | Dense, not filamentous |
|  | Grading of adhesions |
| 0 | None |
| 1-2 | Slight |
| 3-4 | Moderate |
| 5-6 | Severe |
